# In Situ Diagnosis of Scabies Using a Handheld Digital Microscope in Resource-Poor Settings—A Proof-of-Principle Study in the Amazon Lowland of Colombia

**DOI:** 10.3390/tropicalmed3040116

**Published:** 2018-11-02

**Authors:** Hollman Miller, Julian Trujillo-Trujillo, Hermann Feldmeier

**Affiliations:** 1 Public Health Service, Vaupes Department, Mitú 97001, Colombia; hollmanmiller@gmail.com; 2 Department of Neglected Tropical Diseases, Ministry of Health and Social Protection, Bogotá 110311, Colombia; trujillotrujillojulian@gmail.com; 3 Charité—University Medicine Berlin, corporate member of Freie Universität Berlin, Humboldt-Universität zu Berlin and Berlin Institute of Health, 12203 Berlin, Germany

**Keywords:** scabies, diagnosis, digital handheld microscope, resource-poor setting, Amerindian communities, Amazon lowland

## Abstract

Scabies is a neglected tropical disease associated with important morbidity. The disease occurs worldwide and is particularly common in resource-poor communities in the Global South. A validated technique for the diagnosis of scabies in resource-poor settings does not exist. The objective of the study was to determine the practicability and accuracy of handheld digital microscopy in three indigenous communities in the Amazon lowland of Colombia, where scabies is the most common parasitic skin disease. One-hundred-and-eleven children and adults from three indigenous communities with a presumptive diagnosis of scabies were examined clinically by using a handheld digital microscope placed directly on the skin. The microscopical identification of a mite was verified by an “experienced mother”, a woman who had acquired the skills to diagnose scabies as part of traditional Amerindian medicine. The “experienced mother” removed the parasite with a fine needle and placed it on a flat surface in order to enable its direct examination with the digital microscope. Using digital microscopy, scabies was diagnosed in 24 out of 111 participants and confirmed by the extraction of a *Sarcoptes* mites from the acarine eminence. A characteristic tunnel (burrow) with or without mite could be clearly identified irrespective of the degree of pigmentation of the skin. Besides, digital microscopy revealed pathological characteristics of scabies hitherto unknown and impossible to be seen in dermoscopy, such as partially or totally obliterated tunnels, tunnels with multiple entry or exit points, circumscribed hyperpigmentation around obliterated tunnels and mites secluded in a nodule. This proof-of-principle study demonstrated the accurate diagnosis of scabies by handheld digital microscopy in patients with pigmented skin and the feasibility of this technique in resource-poor settings.

## 1. Introduction

Scabies, a parasitic skin disease caused by the mite *Sarcoptes scabiei* var. *hominis*, causes considerable morbidity through both direct effects and secondary bacterial infection [1,2,3,4]. Scabies provokes intense itch, severely affecting sleep and quality of life [5,6]. The disease occurs worldwide and is particularly common in resource-poor communities in the countries of the Global South [7,8]. In developing countries, children bear the highest disease burden, with an average prevalence of 5–10% [3,5]. In countries with a tropical climate, prevalences are up to 25% in the general population with >40% in some communities in the South Pacific and northern Australia [8,9,10,11].

As scabies can mimic a spectrum of skin diseases of infectious and non-infectious etiology, its clinical diagnosis requires experience. Clinical manifestations may differ between babies, children and adults [12,13], but also may vary between settings [14,15] and in the tropics, bacterial superinfection can further complicate the diagnosis [4,8,16]. How to diagnose scabies best in resource-poor settings in endemic areas is still a matter of debate [17]. Usually, the diagnosis is based on a case definition with unknown specificity and sensitivity [3,18]. To enable effective case management in the countries of the Global South, a diagnostic method is needed which is accurate and appropriate in people with moderately to intensely pigmented skin.

*Sarcoptes* mites can only be identified with certainty using a substantial magnification. We, therefore, decided to use a handheld digital video microscope allowing a magnification of up to 200-fold which, e.g., enables to reliably detect movements of the intestine and contractions of the heart of another skin parasite, *Tunga penetrans* [19]. This parasite is embedded in the lower strata of the epidermis and is difficult to identify even in unpigmented skin. The same technique was also used to diagnose myiasis caused by *Dermatobia hominis*, and trombiculid chigger mites (Hollman Miller, unpublished observation 2017). Here we show that (i) *Sarcoptes* mites can be reliably detected inside the tunnel they have created; (ii) tunnels vary considerably in shape, length and structure; and (iii) if a mite is present, it is almost always located in the acarine eminence at the end of the tunnel (burrow).

## 2. Material and Methods

### 2.1. Study Area and Population

The study was performed in Vaupes Department in the Amazon lowland of Colombia. The department has a surface of about 54,000 km^2^. The population mainly consists of indigenous people of various ethnicities. They live in small communities along the Vaupes, Apaporis and Isana River and its affluents. Vaupes is covered with dense rain forest and most communities are only accessible by boat. Mitú, the capital of Vaupes Department, is situated at Latitude: 1°15′28″ North and Longitude: 70°14′04″ West with an altitude of 158 m above sea level. The climate is tropical the whole year round.

Scabies is the most common skin disease in the Amazon lowland of Colombia. Prevalence is in the order of 2% in the general population and 80% of the cases are children (Hollman Miller, unpublished observation 2017). Other important parasitic skin diseases are tungiasis, myiasis and cutaneous leishmaniasis. Prior to the present study, no control measures for scabies were undertaken in the area.

### 2.2. Study Design and Data Collection

The study was performed in the indigenous communities Cariya (N 0°22′22″, W 70°07′46.6″) and Santa Catalina (N 0°21′51.55″, W 70° 5′52.80″), situated along the Tiquié river, as well as in Barrio 12 de octubre, at the periphery of Mitú (N 01°14′00.4″, W 70°14′01.9″) between January and May 2017. Communities were selected on the basis of information from local health personnel that scabies seemed to be frequent and that they were accessible from Mitú by boat or car. In each community, individuals with a presumptive diagnosis of scabies were eligible, independent of their age.

**Figure 1:**
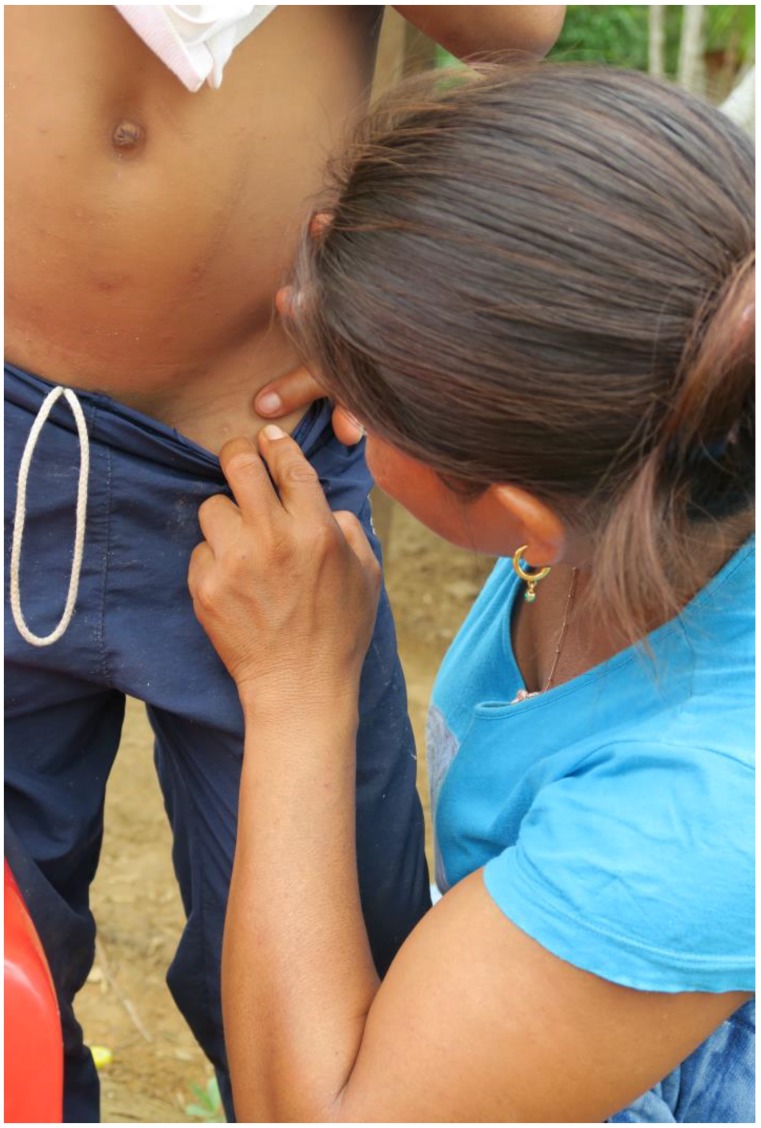
Experienced mother examining the skin of a child for the presence of a pearly vesicle.

**Figure 2:**
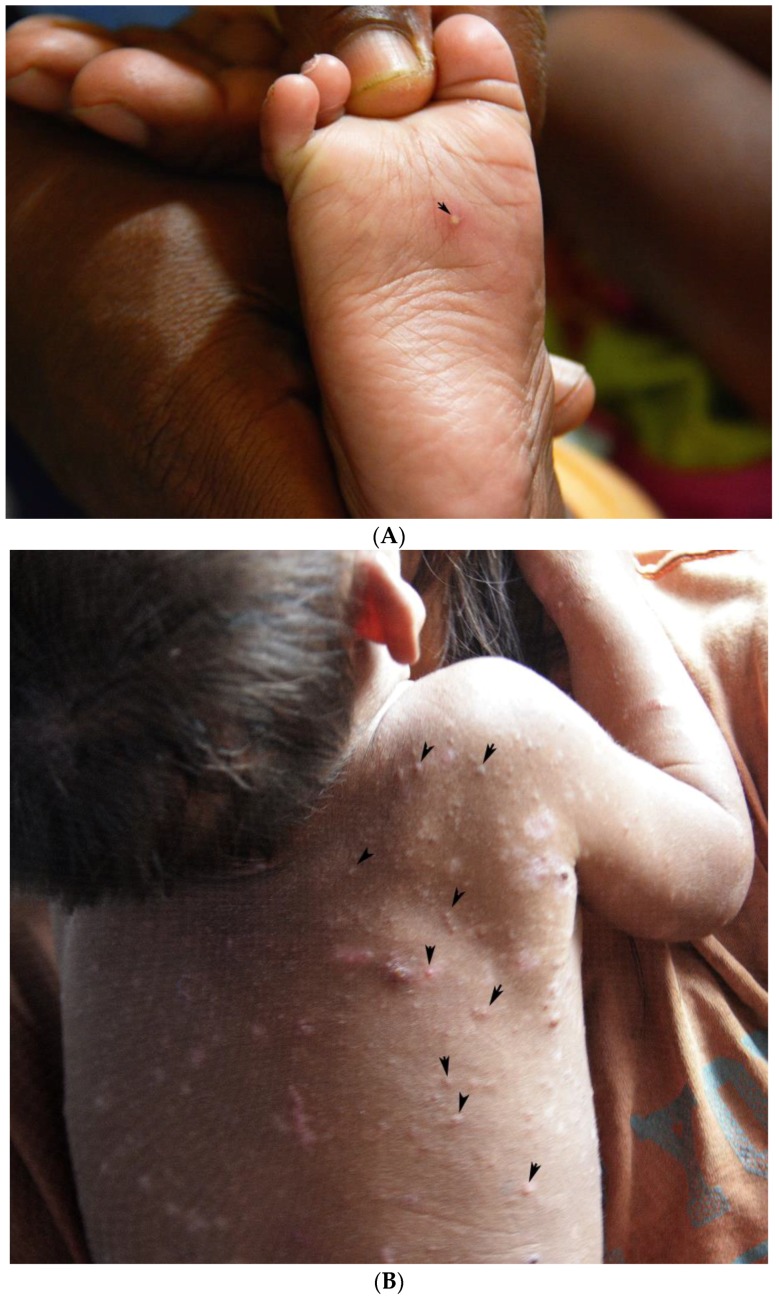
(**A**) A pearly vesicle at the sole of the foot (arrow). (**B)**. Multiple pearly vesicles on the back of a child with severe scabies (arrows).

**Figure 3:**
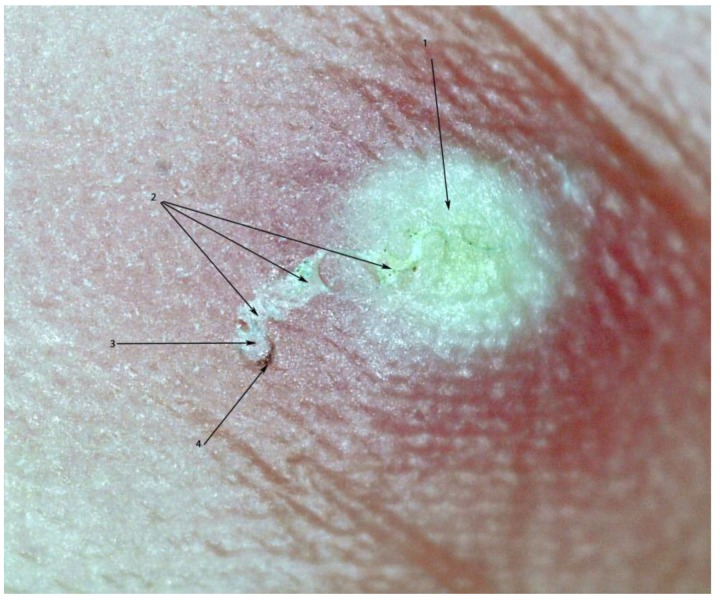
Pearly vesicle (1) and acarine eminence linked by a tunnel (2). The mite is situated at the end of the tunnel with its dorsal part turned towards the surface of the skin (3). The delta-wing sign is clearly visible (4). Photo by digital microscopy; magnification 30-fold.

In indigenous communities in the Amazon lowland of Colombia, scabies is traditionally diagnosed and treated by an experienced mother (“mujer experimentada”). These are women without medical training who have learned to diagnose scabies with the naked eye as a part of traditional Amerindian medicine, usually from their mothers. The experienced mother carefully inspects the patient’s skin and looks for the presence of a pearly vesicle (*vesicula perlada*) which reflects the entry point of a mite into the skin [20] (Figure 1). Pearly vesicles are whitish and translucent, have a diameter of 0.5 to 3 mm and have a soft resistance against pressure (Figure 2A,B). They are frequently decapitated by scratching and then look like an excoriation. Once the experienced mother has identified a pearly vesicle or its remains, she looks for the acarine eminence (*eminencia acarina*), an oval elevation of the skin with a pearlescent appearance at the limit of visibility (0.3 × 0.4 mm) [20]. By experience, the experienced mother knows that the mite is located in the acarine eminence, usually at short distance to a pearly vesicle (Figure 3). When an experienced mother has identified an acarine eminence, she removes the mite from the corneal layer with the help of a sharp thorn or a sharpened piece of hard wood—a method performed in the Americas for hundreds of years [21] {We had very severe itching in the knuckles and on the back of our hands. The missionary told us, these were *Aradores* (farmers) that dig into the skin. (…) They sent for a mulatto woman who prided herself to be able to [diagnose and heal] all the little beasts that dig into man’s skin, the Nigua, the Nuche, the Coya and the Arador; (*Sarcoptes* mite) She heated the tip of a small splinter of very hard wood by the lamp and pierced it in the furrows visible on the skin. After a long search she announced (…) that there was already an *Arador*. I saw a small round sack at the top of the splinter, (…). Because I had the skin full of *Aradores* on both hands, I ran out of patience with the operation, which had lasted well into the night. (…). (Von Humboldt, Südamerikanische Reise, Ullstein Publisher, Berlin, reprint of original, 1979, p. 348)} Hence, in traditional Amerindian medicine, the identification of the acarine eminence is the prerequisite for treatment of scabies.

**Figure 4:**
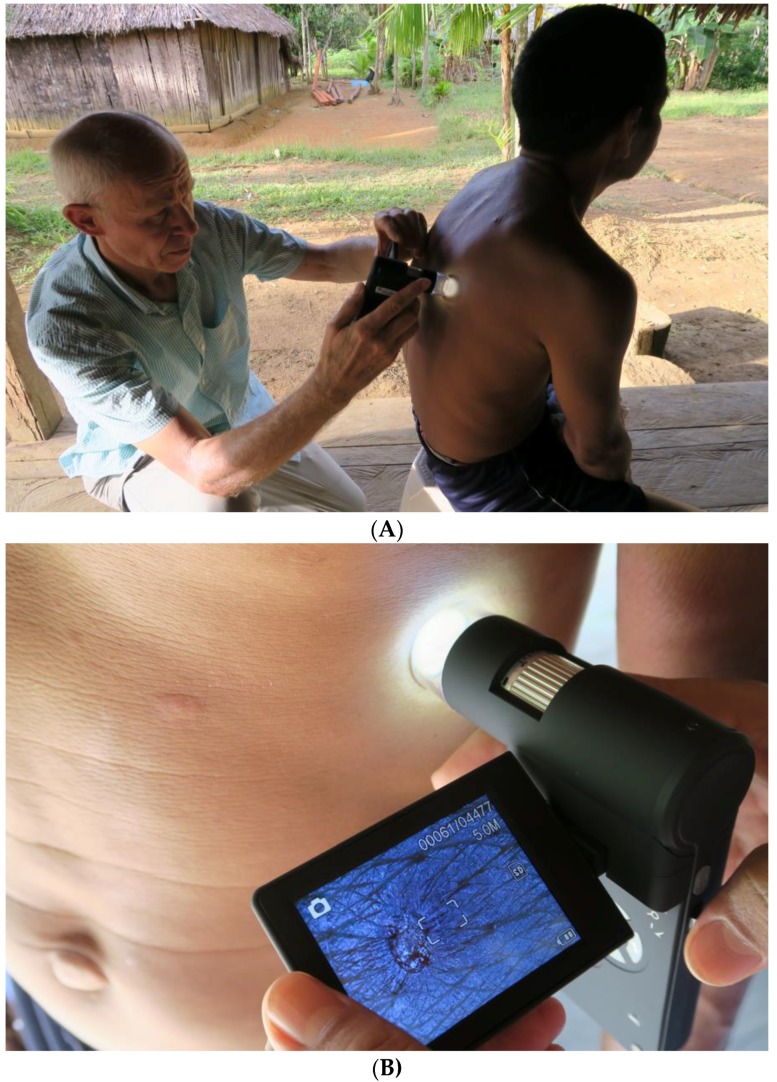
(**A**) Examination of the skin with the digital handheld microscope. (**B**) Examination of the skin with the digital handheld microscope, close-up.

Community health workers were informed about the arrival of the health team a couple of days in advance. Individuals with itchy lesions of the skin were asked to present themselves at a designated place in the morning and in the afternoon. No active search for cases was performed. After the medical history had been taken, the skin was examined macroscopically. Thereafter, the digital handheld microscope (dnt DigiMicro Mobile 5 Megapixel handheld microscope; Drahtlose Nachrichtentechnik GmbH, Dietzenbach, Germany) was placed directly on the skin and suspicious lesions were examined one by one, first using a 10-fold magnification followed by 30-fold magnification to identify the morphological characteristics of *Sarcoptes* mites (Figure 4A,B). Each lesion was scrutinized for the presence of a tunnel (intact, in the process of obliteration or totally obliterated), the presence of a mite inside a tunnel as well as the presence of faecal pellets or eggs. Other characteristics systematically looked for were the acarine eminence and the pearly vesicle [22,23], the presence of coagulated blood inside or around a tunnel (as an indicator of micro-haemorrhage induced by scratching); exfoliation or desquamation of the corneal layer (as an indicator of repeated scratching); and circumscript hyper-pigmented areas, visible only microscopically.

Pathological findings were photographed, stored on an SD-card inside the microscope and transferred to a computer at the end of the day.

**Figure 5:**
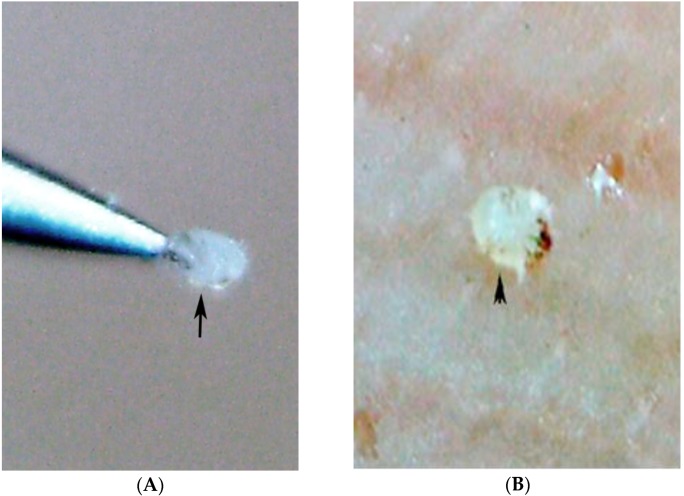
(**A**) *Sarcoptes* mite being extracted with a sterile needle by an experienced mother; magnification 70-fold. (**B**) Dorsal view of a mite extracted by the experienced mother from an acarine eminence; the hyperpigmented area of the anterior part of the mite corresponds to the delta-wing sign as seen in the dermatoscope; magnification 90-fold.

When a mite was detected in an acarine eminence by digital microscopy, the “experienced mother” was asked to remove it with a sterile entomological spin of #3 size and to place it on flat surface, such as a finger nail (Figure 5A,B) to confirm microscopy results. Using the video function of the microscope it was attempted to identify mites moving inside a tunnel or on the surface of the skin.

### 2.3. Diagnosis

Scabies was suspected if a patient showed a suspicious skin alteration accompanied by itching for at least 2 weeks. The following skin alterations were considered suspicious: presence of characteristic primary lesions (papules, crusted papules, vesicles, nodules) with or without the presence of secondary lesions (excoriation of the skin, secondary bacterial infection) that were obviously not associated with other dermatological conditions. Bacterial superinfection was diagnosed when pustules, suppuration or an abscess were present.

Diagnosis of scabies was defined as detection of the morphological characteristics of a mite in an acarine eminence by digital microscopy and subsequent confirmation by the extraction of the parasite; or if faecal pellets were identified inside a tunnel by digital microscopy.

### 2.4. Treatment

After diagnosis, the patient was treated with 200 µg/kg ivermectin orally, followed by a second dose after 1 week. Children <5 years and pregnant and lactating women were treated topically with sulfur in petrolatum 8%, once a day for 3 days [23]. Ivermectin and sulfur in petrolatum are standard treatments of scabies recommended by the Colombian Ministry of Health.

## 3. Results

The demographic characteristics of the study participants are summarized in Table 1. All participants had pigmented skin. Scabies was diagnosed in 24 out of 111 participants (21.6%). The frequency of cases was highest in Cariya (54.5%), and considerably lower in Santa Catalina and Barrio 12 de Octubre (15.6 and 20.4% of study participants, respectively). Sixteen of the 24 patients (66%) were children younger than 10 years of age. The clinical characteristics of the patients are depicted in Table 2.

**Figure 6:**
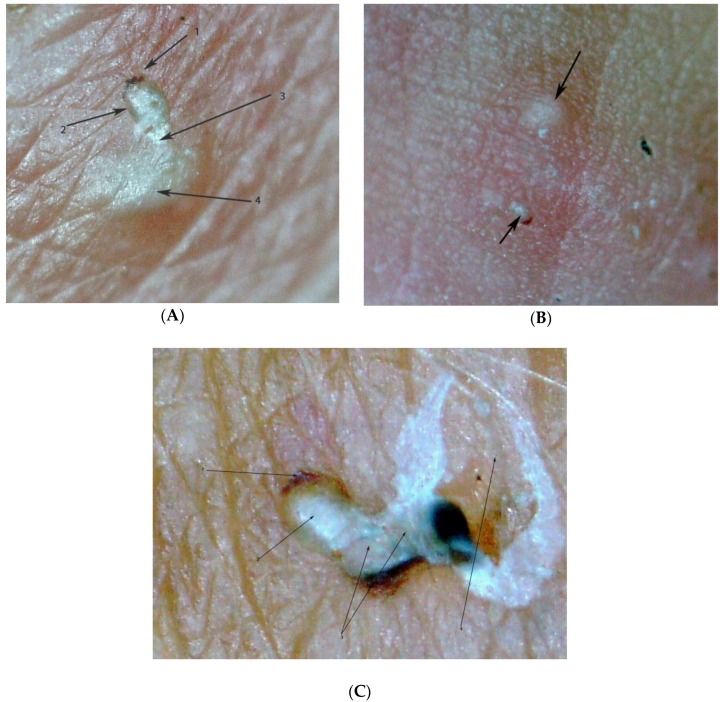
(**A**) Oval-shaped acarine eminence at the end of the tunnel (2) with the delta-wing sign (1); partially obliterated tunnel (3) connecting the acarine eminence with the pearly vesicle (4); magnification 50-fold. (**B**) Pearly vesicle (top) and acarine eminence (bottom) located at a distance from each other; no tunnel visible; the delta-wing sign is clearly visible (arrow bottom); magnification 15-fold. (**C**) Acarine eminence (3) at the end of an S-shaped tunnel containing a mite (2) with delta-wing sign visible (1); pearly vesicle destroyed by scratching (4); magnification 80-fold.

In 23 cases, a mite was identified in an acarine eminence. In one case, a mite was detected in the middle of a tunnel without an acarine eminence (Table 3). Pearly vesicles were observed in 17 cases, usually in a short distance of a few mm to an acarine eminence (Figure 6A,B). Frequently, only the residues of a pearly vesicle were visible (Figure 6C). 

**Figure 7:**
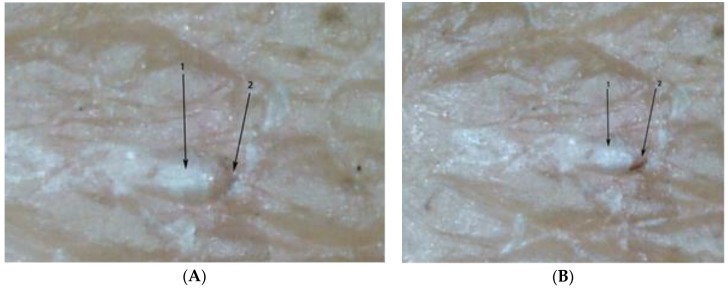
(**A**) Sarcoptes mite inside an acarine eminence with its dorsal part turned towards the surface of the skin (1); delta-wing sign visible (2); magnification 65-fold. (**B**) The same mite after having changed its position (1); now the ventral part is turned towards the surface of the skin (2); delta-wing sign not clearly visible.

**Figure 8:**
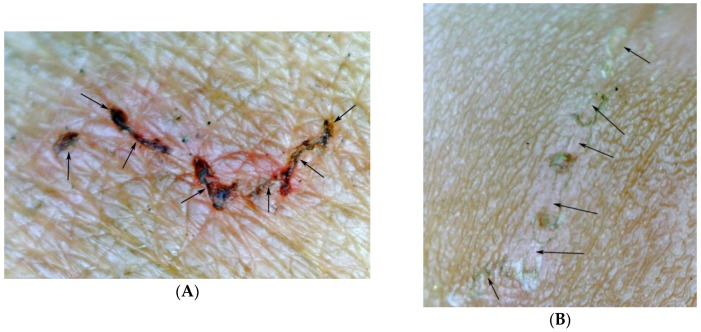
(**A**) Partially obliterated tunnel, obliterated parts show micro-haemorrhages (arrows); magnification 15-fold. (**B**) Totally obliterated tunnel; only residues of micro-haemorrhages visible; magnification 15-fold.

**Figure 9:**
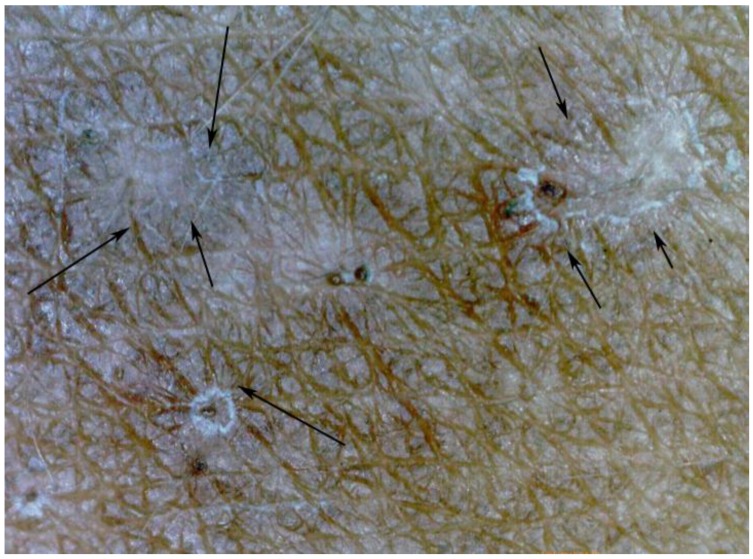
Microscopic circumscribed hyperpigmented areas (arrows) around a sinusoidal tunnel; magnification 30-fold.

**Figure 10:**
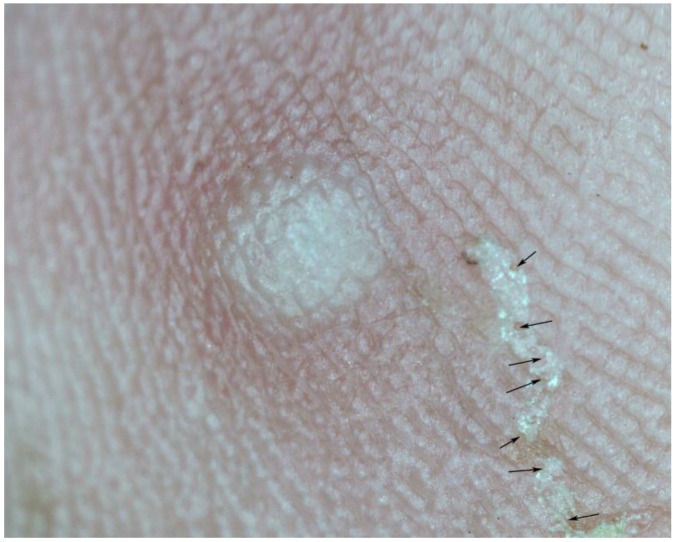
Serpiginous tunnel with multiple entry and exit points (arrows); magnification 15-fold.

**Figure 11:**
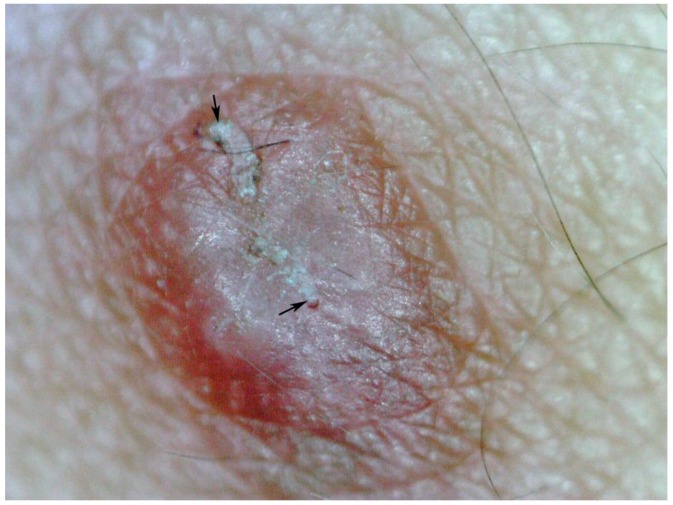
Nodule containing two mites (arrows); magnification 25-fold.

The microscopic aspect of a mite differed according whether its ventral or dorsal part was turned towards the surface of the skin (Figure 7A,B). Partially or completely obliterated tunnels were observed in 16 patients (Figure 8A,B). Those tunnels did not contain a mite. Tunnels in the process of obliteration showed a circumscript hyperpigmentation, not visible macroscopically (Figure 9). The length of a tunnel varied from less than 1 mm to about 10 mm. In some cases, tunnels showed multiple entry and exit points (Figure 10). In a single case, a mite was detected outside a tunnel moving on the skin. One nodule was identified which contained 2 mites (Figure 11).

**Figure 12:**
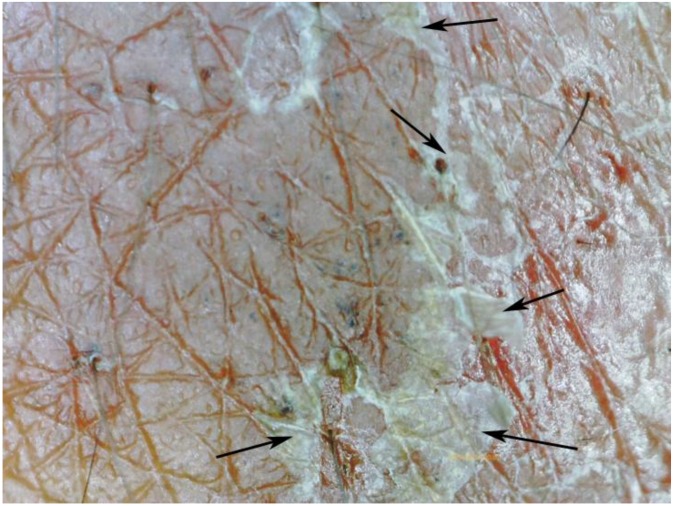
Microscopic excoriations of the stratum corneum not visible microscopically (arrows); magnification 100-fold.

Usually, when excoriations were present macroscopically, micro-hemorrhages were visible microscopically (Figure 8A). Besides, the handheld digital microscope demonstrated the presence of tiny excoriations which were not visible macroscopically (Figure 12).

**Figure 13:**
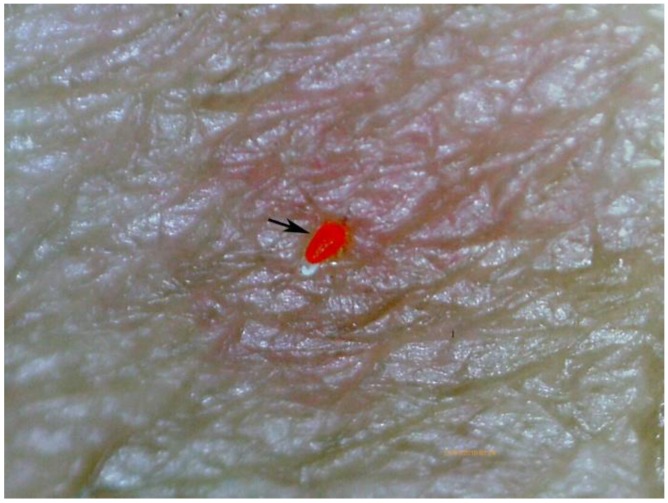
*Trombicula alfreddugesi* identified by the digital handheld microscope (arrow); magnification 10-fold.

Lesions caused by *Trombicula alfreddugesi*, a mite endemic in the tropical rain forest of the Amazon and the Orinoco Basin, were ancillary findings by digital microscopy. The morphological characteristics of *T. alfreddugesi* are easily differentiated from those of a *Sarcoptes* (Figure 13).

All participants of the study were at ease with the examination of the skin by digital microscopy. Elder patients found it informative to spot the parasite that caused their disease. 

## 4. Discussion

Scabies can mimic a broad range of infectious and non-infectious diseases [12] and the clinical picture is frequently masked by superinfection [13]. Hence, the specificity of clinical diagnosis is low, and its sensitivity depends on the experience of the observer. Since simple and accurate diagnostic techniques do not exist, in resource-poor settings, the diagnosis of scabies is based on the presence of suspicious clinical findings such as itchy papules or on a case definition of which neither sensitivity nor specificity is known [18,24].

Our findings also explain why a diagnosis based solely on the identification of a tunnel, such as by means of a burrow ink test, has a notoriously low sensitivity and specificity [25,26].

Based on our findings in tungiasis, a skin disease where the parasite is embedded in the lower strata of the epidermis and is difficult to identify macroscopically [19], we decided to use a digital handheld microscope for the detection of *Sarcoptes* mites in situ. The application of the digital handheld microscope is totally inoffensive and well tolerated even by small children [19]. The digital microscope is directly placed on the skin and illuminates a circumscript area of the skin of a diameter of 30 mm with 8 LED. The area examined is displayed on a color monitor with a resolution of 5 megapixels. Resolution of pictures can be further enhanced by connecting the microscope with a laptop or a tablet computer and enlarging the picture on the computer in real time. Photos and videos are stored on a Micro-SD card inside the microscope. The price of the handheld digital microscope is in the order of 120 US$. Previously described optical methods for the in vivo detection of mites used highly sophisticated stationary and expensive equipment such as reflectance-mode confocal microscopy or epiluminescence microscopy which are not suitable for resource-poor settings [27,28,29].

The digital handheld microscope allowed the identification of 24 cases of scabies out of 111 participants who fulfilled the admission criteria. Except for one case, mites were seen in the acarine eminence at the end of the tunnel. We presume that the acarine eminence is caused by a mite feeding on corneal cells and thereby exerting pressure on the surrounding layers of corneal cells. It reflects a dilatation of the upper corneal layer by a biologically active foreign body moving forward in the skin. The pearly vesicle was described first by the French dermatologist Pierre-Antoine-Ernest Bazin in 1862 [22]. We suppose that the pearly vesicle reflects an acute local inflammation at the point where a female mite has penetrated into the stratum corneum. Due to scratching, a pearly vesicle is frequently decapitated and only a residue remains. Ideally, the pearly vesicle and the acarine eminence should be linked by a tunnel. In practice though, pearly vesicles and tunnels are destroyed by scratching or become invisible due to bacterial superinfection [20,23]. The appearances of the pearly vesicle may vary under different environmental conditions and in colder climates and older persons, for instance, the mite entry point is often drier and scaly. This emphasizes the importance of adapting training to suit local conditions and using descriptive words that are easily understandable by local health workers.

An intriguing observation was the finding that the ventral and the dorsal part of an embedded *Sarcoptes* mite showed a different pigmentation pattern: The triangular pigmentation at the anterior part of the mite (the delta-wing sign visible in dermoscopy) being pronounced, when its dorsal part was turned towards the skin. Since the other body areas of a mite are almost translucent and a mite may change its position with regard to the skin’s surface, this explains why in dermoscopy the delta-wing sign is not consistently present [24,30,31,32]. Obviously, even in non-pigmented skin, the delta-wing sign cannot be seen in the dermatoscope when the ventral end of the mite is positioned towards the surface of the skin or when the mite has changed its direction and its rear end is situated towards the end of the tunnel.

Faecal pellets were only inconsistently detectable in the handheld digital microscope and eggs were only seen in a single occasion. Neither faecal pellets nor eggs are reliable indicators of active scabies in digital microscopy and dermoscopy. Actually, it has been previously shown that eggs and faecal pellets need to be stained to become clearly visible [33,34].

The digital microscope also showed that tunnels considerably vary in size and shape and degree of obliteration. Surprisingly, we observed that tunnels may have various entry and exit points and that there are intact tunnels not inhabited by a mite. This confirms previous observations [29]. The presence of a tunnel as such, therefore, cannot be considered pathognomonic for the presence of active scabies and does not allow for a conclusion on the necessity of treatment [28]. Our findings also explain why a diagnosis based solely on the identification of a tunnel, such as by means of a burrow ink test, have a notoriously low sensitivity and specificity [25,26].

Our study corroborates previous findings that dermoscopy is an inappropriate means to diagnose scabies in a resource-poor setting [24]. Although the sensitivity of the technique is rather high (0.85; 95% CI 0.70–0.94), its specificity is low (0.46; 95% CI, 0.34–0.58) [24]. This can be explained by the finding that dermoscopy only yields optimal results when examiners are trained in the diagnosis of scabies [30] and that artefacts induced by scratching, such as crusts, or punctuate bleeding or small particles of dirt can be confounded with a mite [35,36].

Our study has several limitations. First, due to the lack of privacy, we excluded the genital area in both sexes and the peri-mamillary area in women. Since the morphological characteristics of lesions at these topographic areas may differ from those of other parts of the skin, e.g., noduli are very common on the penis, but rare at the abdomen, we cannot exclude that the distribution patterns of acarine eminence and/or the pearly vesicle are the same at all topographic areas of the skin. Second, we used the extraction of a mite from the acarine eminence by the experienced mother as a proof that the morphological characteristics seen in the digital handheld microscope were actually that of a *Sarcoptes* mite. We therefore can only conclude on the specificity of the method, but not on its sensitivity. A study to compare the sensitivity of the digital handheld microscopy with other diagnostic techniques used in resource-poor settings is currently being performed. Third, since all patients presented themselves voluntarily, we cannot rule out that a selection bias towards patients with a severe form of scabies might have occurred.

In conclusion, the proof-of-principle study showed that the digital handheld microscope is an accurate diagnostic instrument to diagnose scabies in resource-poor settings in people with pigmented skin. In addition, the technique allows the detection of micro-pathological alterations of the skin presumably caused by scratching or due to an immune response of the host. If applied in a systematic way, handheld digital microscopy will provide new insights into the biological host-parasite-relationships of *Sarcoptes* mites and eventually lead to the understanding of pathogenesis and pathophysiology of skin alterations caused by *S. scabiei*.

## Figures and Tables

**Table 1:** Demographic characteristics of participants.

Community	Number of Participants	Age in Years Median (Range)	Males/Females	Scabies Diagnosed ^a^ (%)
Cariyá	11	29 (3–88)	4/7	6 (54.5)
Santa Catalina	51	19 (2–71)	28/23	8 (15.7)
Barrio 12 de Octubre	49	12 (3 months–77)	21/28	10 (20.4)
Total	111	15 (3 months–88)	53/58	24 (21.6)

^a^ see Material and Methods.

**Table 2:** Clinical characteristics of 24 patients with scabies.

Characteristics	Frequency (n/%)
Number of topographic areas affected	
1–5	10/(41.6%)
6–10	13/(54.1%)
>10	1/(4.31%)
Appearance of lesions (weeks ago)	
<4 weeks	6/(25.0%)
4–12 weeks	14/(58.3%)
>12 weeks	4/(16.6%)
Type of lesion ^a^	
Papule	23/(95.8%)
Vesicle	16/(66.6%)
Nodule	2/(8.3%)
Crusted lesion ^b^	15/(62.5%)
Excoriation/desquamation	19/(79.1%)
Bacterial superinfection ^c^	15/(62.5%)

^a^ Multiple classifications possible. ^b^ Crust developed on top of vesicle or papule. ^c^ Pustule, suppuration.

**Table 3:** Findings by digital microscopy in 24 scabies patients ^a^.

Characteristics ^b^	Frequency (n/%)
Tunnel without mite	8/(33.7%)
Tunnel with mite	24/(100%)
Tunnel with mite present in the acarine eminence	23/(95.8%)
Tunnel with mite in the middle of the tunnel	1
Tunnel with faecal pellets	8/(33.3%)
Partially or totally obliterated tunnel	16/(66.7%)
Extraction of mite by experienced mother	24/(100%) ^c^
Pearly vesicle surrounded by erythema	17/(70.8%)
Circumscribed hyperpigmentation	22/(91.6%)
Micro-haemorrhagia	4/(16.6%)
Excoriation/desquamation of the corneal layer	19/(79.1%) ^d^
Nodule containing mite	1/(4.1%)

^a^ For case definition see Materials and Methods ^b^ Multiple classifications possible. ^c^ In four cases, the mite was lost directly following the extraction. ^d^ not visible macroscopically.
